# PARP inhibitors in the management of breast cancer: current data and future prospects

**DOI:** 10.1186/s12916-015-0425-1

**Published:** 2015-08-13

**Authors:** Luca Livraghi, Judy E. Garber

**Affiliations:** grid.65499.370000000121069910Department of Medical Oncology, Dana-Farber Cancer Institute, Boston, MA 02215 USA

**Keywords:** BRCA, Breast cancer, PARP inhibitors, Poly(ADP-ribose) polymerases

## Abstract

Poly(ADP-ribose) polymerases (PARP) are enzymes involved in DNA-damage repair. Inhibition of PARPs is a promising strategy for targeting cancers with defective DNA-damage repair, including *BRCA1* and *BRCA2* mutation-associated breast and ovarian cancers. Several PARP inhibitors are currently in trials in the adjuvant, neoadjuvant, and metastatic settings for the treatment of ovarian, *BRCA*-mutated breast, and other cancers. We herein review the development of PARP inhibitors and the basis for the excitement surrounding these agents, their use as single agents and in combinations, as well as their toxicities, mechanisms of acquired resistance, and companion diagnostics.

## Background

Modern strategies for the development of novel cancer therapies include agents targeting specific molecular defects that characterize certain cancer cells in order to increase treatment efficacy and reduce toxicities. In breast cancer, targeted therapies have long been effective, as agents targeting hormone receptors in tumors expressing them and as antibodies or tyrosine kinase inhibitors targeting overexpressed or amplified HER2 molecules. Breast tumors expressing none of these are called triple-negative breast cancers (TNBC), which comprise about 15 % of breast cancers overall, about 70 % of breast cancers in individuals harboring a germline *BRCA1* mutation, and 20 % in *BRCA2* mutation carriers [1–4]. The discovery of the family of nuclear enzymes poly(ADP-ribose) polymerases (PARPs) and their role in DNA-damage repair pathways opened the possibility of developing a new class of antineoplastic drugs with the ability to interfere with the DNA damage repair systems of cancer cells – PARP inhibitors (PARPi). One characteristic of *BRCA*-mutated cancers is defective function of one of the major DNA damage repair pathways, the homologous recombination (HR) pathway. The original concept of the activity of PARP inhibitors was that they acted through synthetic lethality by targeting the base excision repair pathway (BER); in tumor cells with defects in a different DNA repair mechanism, disruption of both pathways led to cell death. The preferential sensitivity of BRCA-associated breast and ovarian cancers was therefore predicted as the tumor cells are characterized by defective homologous recombination repair. Subsequently, PARPi have shown significant activity in BRCA-associated breast, ovarian, and other cancers [5, 6]. However, the activity in sporadic ovarian cancers suggests a more complex mechanism of action described below [7].

### PARPs and DNA damage repair

PARPs are a family of enzymes involved in various activities in response to DNA damage [8]. Eighteen components of this family have been discovered; PARP-1 to -3 are so far the only members defined as DNA damage-dependent PARPs [9].

**Fig. 1:**
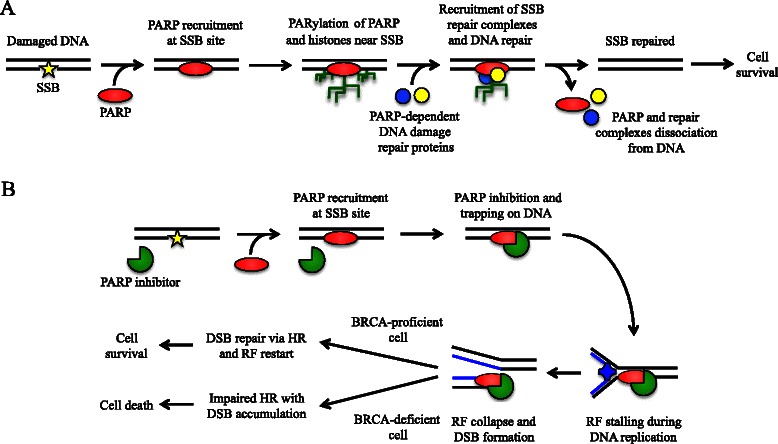
Current model for PARP role in DNA damage repair and PARP inhibition – BRCA mutation synthetic lethality. **a** When single-strand break (SSB) is detected, PARP recruitment and activation leads to SSB repair through poly(ADP-ribosyl)ation (PARylation) of histones and chromatin remodeling enzymes, auto-PARylation of PARP, and recruitment of PARP-dependent DNA repair proteins. Repaired DNA can undergo replication determining cell survival. **b** In the presence of PARP inhibitors, PARPs recruited to DNA-damage sites are no longer able to activate PARP-dependent repair systems and to dissociate from DNA (due to catalytic activity inhibition and/or direct trapping), determining replication fork (RF) stalling during DNA replication. Stalled RF eventually collapse creating double strand break (DSB). DSB can be repaired by homologous recombination (HR) and replication may restart, leading to cell survival. In BRCA-deficient cells, HR is impaired, thus DSB cannot be efficiently repaired; in this context, DSB accumulate determining cell death

PARP activation, largely driven by DNA damage (other mechanisms may occur, as reviewed by Bürkle et al. [10]), determines post-transcriptional modification of nuclear proteins such as histones [9]. PARP-1 activation is one of the earliest responses to DNA damage in human cells [11, 12]. The ADP-ribosylation of histones and the recruitment of chromatin remodeling enzymes create a relaxed chromatin state that is appropriate for DNA repairing activities (Fig. 1a). The ADP-ribose polymer synthesized by PARP acts as a “flag” that drives the assembly of DNA-repair complex at sites of DNA damage, mainly promoting BER and single strand break repair (SSBR) pathways [9], while involvement of PARPs in double strand break repair (DSB – an error-free DNA repair system) is likely limited [13].

### Rationale for development of PARPi in breast cancer

Since cancer is a disease in which DNA replication is critical, replication errors are prominent, and deficiencies in DNA-repair pathways are common [14], the involvement of PARPs in DNA-repair pathways stimulated the development of agents capable of targeting PARP activity.

To maintain DNA integrity, HR-deficient cells rely on secondary DNA repair pathways, such as BER, SSBR, and non-homologous end joining. When PARP-dependent activation of BER/SSBR and non-homologous end joining is defective, cells rely on the HR pathway to restore DNA integrity. BRCA1 and BRCA2 proteins are key actors of the HR apparatus and deficiency of either (secondary to germline mutation in one copy and loss of heterozygosity inactivating or removing the other copy) results in inefficient activation of HR (Fig. 1b). Using *BRCA1*- and *BRCA2*-deficient cell lines and mouse xenografts, Bryant et al. [15] and Farmer et al. [16] demonstrated marked *in vitro* and *in vivo* cytotoxicity of PARPi monotherapy in tumor cells with intrinsic HR deficiency, with close to no effect on BRCA-proficient cells.

The model explaining this “synthetic lethality” effect of PARP inhibition in HR-deficient cells is comprehensively reviewed by Helleday [17]. Briefly, suppression of PARP catalytic activity blocks the formation of ADP-ribose polymers at site of SSB, hence PARP-dependent DNA-damage repair complexes cannot be efficiently recruited. Unrepaired SSB eventually lead to stalling of replication forks [17]. Stalled replication forks collapse into double strand breaks that are highly cytotoxic lesions if not repaired by HR [17], the repair mechanism inefficiently activated in *BRCA*-mutated cancers. Recent data suggest that another mechanism of action of PARPi, so-called “PARP trapping”, is more important in determining PARPi cytotoxicity. Murai et al. [18] showed that PARPi prevent dissociation of recruited PARPs from DNA-damage sites: these stabilized PARP/DNA complexes determine stalling of the replication fork during DNA replication, with subsequent formation of double strand breaks.

The observation that *BRCA*-mutated breast cancers show an impairment in HR pathways [19], and that some sporadic TNBC are phenocopies of *BRCA1*-mutated cancers (i.e. they display a phenotype resembling *BRCA1*-mutated cancers without harboring a *BRCA1* mutation, a feature also defined as “BRCAness”, see below) [20, 21], led to exploration of the application of PARP inhibition to the treatment of breast cancer (BRCA-associated and TNBC).

### Clinical application in breast cancer

Clinical development of PARPi started in 2003 and focused on two strategies: utilizing PARPi in combination with other drugs in a range of solid malignancies or using PARPi monotherapy in specific cancer types with features (like impairment of DNA-damage repair systems alternative to the PARP-dependent ones) that would be predicted to be highly sensitive to PARP inhibition. Testing of PARPi in combination with cytotoxic drugs showed the feasibility of this approach with overall good tolerability, but there was little evidence of activity in unselected patients [22]. In contrast, promising data emerged in the treatment of patients with breast and ovarian cancers [23, 24], the two malignancies most frequently associated with *BRCA* mutations.

Clinical testing of PARPi was initially slowed by negative results from a phase 3 trial of iniparib, a compound inaccurately classified as a PARPi [25]. Subsequently, it was shown that iniparib and its metabolites do not inhibit PARP in intact cells [26], and clinical development of genuine PARPi gained new vigor. Currently, five compounds with the ability to inhibit the activity of various PARPs are being investigated in clinical trials (Table 1). Below, we will present the most important findings from phase 1 and 2 clinical trials evaluating the efficacy of PARPi in the treatment of breast cancer. These data are also summarized in Tables 2 and 3.

**Table 1:** PARPi compounds in clinical development

Drug name	Pharmaceutical company	Current investigational phase in breast cancer
Olaparib (AZD2281)	AstraZeneca	Phase 3 studies in adjuvant and advanced settings in germline BRCAm breast cancer
Veliparib (ABT-888)	Abbvie	Phase 3 study in neoadjuvant setting in combination with carboplatin and standard therapy in triple-negative breast cancer
		Phase 2/3 studies in advanced setting as combination therapy in germline BRCAm breast cancer
Niraparib (formerly MK-4827)	Tesaro	Phase 3 study in advanced setting in germline BRCAm breast cancer
Talazoparib (BMN-673)	BioMarin Pharmaceuticals	Phase 3 study in advanced setting in germline BRCAm breast cancer
		Phase 2 studies in advanced setting in BRCAm breast cancer
		Phase 2 study in advanced setting in germline BRCA intact breast cancer
		Phase 2 study in neoadjuvant setting in BRCAm breast cancer
Rucaparib (formerly AG-14699)	Clovis Oncology	Phase 2 study in advanced setting in patients with known germline BRCAm solid tumors
		Phase 2 study in adjuvant setting in triple-negative breast cancer or germline BRCAm breast cancer
CEP-9722	Teva Pharmaceutical Industries	Phase 2 study in advanced setting in solid tumors

BRCAm, *BRCA1*/*2* mutation-associated

**Table 2:** Phase 1/2 studies of PARPi monotherapy in metastatic breast cancers, with spotlight on BRCA mutated patients

Study Name (NCT)	Ref.	Phase	Tumor type	No. of patients	Investigation arm	Primary endpoint	Overall results	Results in BRCAm BC	Additional notes
				[total/BC (BRCAm BC)]					
Olaparib									
NCT00516373	[5]	1	Solid tumors	60/9 (3)	Olaparib (10–600 mg bid)	PK, PD, safety and tolerability	ORR: 15 %	ORR 33 %	One CR in BRCAm BC lasting more than 60 weeks
NCT00572364	[119]	1	Solid tumors	12/4 (NK)	Olaparib (100–400 mg bid)	Safety and tolerability	ORR: 8 %	–	One patient with BC and family history of BC had PR for 13 months
NCT00494234	[34]	2	BRCAm BC	54/54 (54)	C1: olaparib (400 mg bid)	ORR	–	ORR C1: 41 %	
					C2: olaparib (100 mg bid)			ORR C2: 22 %	
NCT00679783	[7]	2	TNBC or BRCAm BC, HGSOC or BRCAm OC	90/26 (10)	Olaparib (400 mg bid)	ORR	ORR in BC: 0 %	ORR: 0 %	Evidence of activity in non-BRCAm OC and platinum-resistant OC
							ORR in OC: 29 %	50 % of unconfirmed PR (by RECIST)	
NCT01078662	[6]	2	BRCAm solid tumors	317/62 (62)	Olaparib (400 mg bid)	ORR	ORR: 26 %	ORR: 13 %	Mean number of previous regimens for advanced disease: 4.6
								DS ≥8 weeks: 47 %	
Veliparib									
NCT00892736	[120]	1	TNBC, HGSOC and BRCAm BC and OC	98/35 (14)	Veliparib (50–500 mg)	Tolerability	ORR in BRCAm: 24 %	ORR: 29 %	
							ORR in BRCA wt: 4 %	CBR: 57 %	
Talazoparib									
NCT01286987	[32]	1	Solid tumors	39/8 (6)	Talazoparib (25–1100 μg)	PK, PD, safety and anti-tumor activity	ORR: 65 % in BRCAm OC	ORR: 33 %	
Niraparib									
NCT00749502	[31]	1	Solid tumors	100/12 (4)	Niraparib (30–400 mg)	Safety and tolerability	ORR: 18 % in overall population, 40 % in BRCAm OC	ORR: 50 %	
Rucaparib									
NCT01482715	[33, 121]	1–2	BRCAm BC and OC	56/27 (27)	Rucaparib (18 mg/m^2^)	ORR	Data mixed between OC and BC, at RP2D ORR: 80 % (4/5)	–	

*BC* Breast cancer, *BID* bis in die, *BRCAm BRCA1*/*2* mutation-associated, *CBR* Clinical benefit rate; *CR* Complete response, *DS* Disease stabilization, *HGSOC* High-grade serous ovarian cancer, *NK* Not known, *OC* Ovarian cancer, *ORR* Objective response rate, *PD* Pharmacodynamics, *PK* Pharmacokinetics, *RP2D* Recommended phase 2 dose, *TNBC* Triple-negative breast cancer, *WT* Wild type

Clinical benefit: CR + PR + SD for ≥24 weeks

**Table 3:** Phase 1/2 studies of PARPi as combination therapy in metastatic breast cancers, with spotlight on BRCA mutated patients

Study name (NCT)	Ref.	Phase	Tumor type	No. of patients	Investigation arm	Primary endpoint	Overall results	Results in BRCAm BC	Additional notes
				[total/BC (BRCAm BC)]					
Olaparib									
NCT00707707	[122]	1	TNBC	19/19 (NK)	Olaparib (200 mg bid) + PTX (90 mg/m^2^)	Safety and tolerability	ORR: 37 %	–	First- or second-line treatment only
NCT00710268	[79]	1	Solid tumors	12/3 (NK)	Olaparib (100–400 mg bid) + BEV (10 mg/kg)	Safety and tolerability	No data on response reported	–	No grade 3 or 4 hematologic toxicities
NCT00782574	[23]	1	BC, OC, peritoneal cancer, pancreatic cancer	54/42 (17)	Olaparib (50–200 mg bid continuously vs. intermittent) + CDDP (75 mg/m^2^)	Safety and tolerability	ORR: 41 %	ORR: 71 %	Continuous olaparib schedules not tolerable (hematologic toxicity)
NCT01116648	[45]	1	TNBC, OC	28/8 (3)	Olaparib (100–400 mg bid) + cediranib (20–30 mg)	Safety and tolerability	Overall ORR: 29 %	ORR: 0 %	
							BC ORR: 0 %		
							BC CBR: 29 %		
NCT01445418	[123]	1	BRCAm OC and BC	45/8 (8)	Olaparib (100–400 mg bid) + CBDCA (AUC 3–5)	Safety and tolerability	ORR: 52 %	ORR: 88 %	One CR in BRCAm BC for 3 months
Veliparib									
NCT00535119	[124]	1	Solid tumors	68/14 (NK)	Veliparib (20–120 mg) + CBDCA (AUC 5–6) + PTX (150–200 mg/m^2^)	PK, safety and tolerability	ORR: 19 %	–	One CR in BC
NCT00740805	[125]	1	Solid and hematologic tumors	18/14 (5)	Veliparib (50–150 mg) + DOX (60 mg/m^2^) + CYC (600 mg/m^2^)	Tolerability	No overall results reported	ORR: 60 %	Expansion cohort study in BC ongoing
NCT01063816	[126]	1	Solid tumors	59/10 (NK)	Veliparib (250 mg bid) + CBDCA (AUC 4) + GEM (800 mg/m^2^)	PK, safety and tolerability	ORR: 22 %	–	
NCT01104259	[53]	1	TNBC or BRCAm BC	45/45 (12)	Veliparib (20–300 mg) + CDDP (75 mg/m^2^) + VNR (25 mg/m^2^)	Tolerability	ORR: 55 %	ORR: 73 %	
NCT01251874	[127]	1	BC	44/44 (16)	Veliparib (50–200 mg) + CBDCA (AUC 5–6)	Safety and tolerability	ORR: 19 %	ORR: 25 %	
NCT01445522	[128]	1	Solid tumors and lymphomas	35/12 (NK)	Veliparib (20–80 mg) + CYC (50 mg)	Safety and tolerability	ORR: 20 %	–	
Not reported	[54]	1	BRCAm BC	26/26 (26)	Veliparib (50–200 mg) + CBDCA (AUC 5–6)	Safety and tolerability	–	ORR: 46 %	
								CBF: 74 %	
NCT01281150	[55]	1	Solid tumors	30/24 (5)	Veliparib (50–200 mg) + PTX (80 mg/m^2^) + CBDCA (AUC 5–6)	Tolerability	ORR: 48 %	ORR: 60 %	ORR in non-mutated BRCA BC: 67 %
NCT01009788	[52]	2	BC	41/41 (8)	Veliparib (40 mg bid) + TMZ (150 mg/m^2^)	ORR	ORR: 13 %	ORR: 50 %	Expansion cohort with additional 20 patients with *BRCA1*/*2* mutations: ORR 15 %, CBR: 45 %
								CBF: 63 %	
Rucaparib									
NCT01009190	[129]	1	Solid tumors	23/5 (NK)	Rucaparib (80–360 mg) + CBDCA (AUC 3–5)	Safety and tolerability	DCR: 50 %	–	
CEP-9722									
NCT00920595	[130]	1	Solid tumors	26/7 (NK)	CEP-9722 (150–1000 mg) + TMZ (150 mg/m^2^)	PK, PD, safety and anti-tumor activity	ORR: 5 %	–	

*BC* Breast cancer, *BEV* Bevacizumab, *BID* bis in die, *BRCAm BRCA1*/*2* mutation-associated, *CBDCA* Carboplatin, *CBR* Clinical benefit rate, *CDDP* Cisplatin, *CR* Complete response, *CYC* Cyclophosphamide, *DCR* Disease control rate, *DOX* Doxorubicin, *DS* Disease stabilization, *GEM* Gemcitabine, *HGSOC* High-grade serous ovarian cancer, *NK* Not known, *OC* Ovarian cancer, *ORR* Objective response rate, *PD* Pharmacodynamics, *PK* Pharmacokinetics, *PTX* Paclitaxel, *RP2D* Recommended phase 2 dose, *TMZ* Temozolomide, *TNBC* Triple-negative breast cancer, *VNR* Vinorelbine, *WT* Wild type

Clinical benefit: CR + PR + SD for ≥24 weeks

Disease control: CR + PR + SD for ≥12 weeks

### Clinical trials in advanced disease

#### PARPi as single agent therapy

Following the demonstration by Bryant and Farmer [15, 16] of the cytotoxic effect of PARP inhibition in HR-deficient cells, there was interest in studying the activity of PARPi as monotherapy in solid tumors. In earlier studies, the population enrolled in these trials was not restricted to patients with known *BRCA* mutations, but encompassed also those whose cancer displayed a phenotype similar to *BRCA*-mutated cancers. Clinically, this group included triple-negative breast cancers and high-grade serous or poorly differentiated ovarian cancer. The term “BRCAness” was introduced to identify sporadic tumors that shared common phenotypic features with familial BRCA tumors [20]. Attempts to identify cancers with BRCAness included evaluation of epigenetic silencing of *BRCA* genes [27], measurement of levels of proteins involved in HR [28], and of foci of DNA-repair proteins like gammaH2AX [5, 29]. However, after preliminary data showing minimal efficacy of PARPi in sporadic breast cancers, some of the trials were amended to enrich the study cohorts for BRCA-associated tumors [5, 30].

Initial phase 1 testing of olaparib as monotherapy in BRCA-associated breast and ovarian cancers showed encouraging results: 47 % of patients with BRCA-associated breast, ovarian, or prostate cancers treated with olaparib achieved a partial response, and 63 % of them derived clinical benefit (tumor marker decrease or radiologic response or stable disease for 4 or more months) [5]. A phase 1 study of niraparib in patients with advanced solid tumors enriched for BRCA-associated cancers reported an overall response rate of 40 % (8 of 20) in patients with BRCA-associated ovarian cancer and 50 % (2 of 4) in patients with BRCA-associated breast cancer [31]. Talazoparib monotherapy has shown antitumor activity in patients with *BRCA* mutations, with an objective response rate of 65 % in ovarian and peritoneal tumors and 33 % (2 of 6 patients) in breast cancers [32]. Data presented at ASCO 2014 on single agent rucaparib showed efficacy in BRCA-associated ovarian, breast, and pancreatic cancers [33].

These data from phase 1 trials guided the development of phase 2 studies in the population of patients with BRCA-associated cancers or with cancer usually associated with “BRCAness”, namely triple-negative breast cancer and high-grade serous ovarian cancer (HGSOC).

Tutt et al. [34] reported efficacy of olaparib as monotherapy in 54 patients with advanced breast cancer and germline *BRCA1*/*2* mutations. At the maximum tolerated olaparib dose of 400 mg bid, a 41 % objective response rate was observed, with responses in both TNBC and hormone receptor-positive HER2-negative patients. Toxicities were generally manageable, with treatment-related adverse events reported in 81 % of patients, but grade 3 or 4 events occurred in only 24 % of patients. Efficacy data from this study compare favorably with response rates in studies of single agent cytotoxics (capecitabine [35], vinorelbine [36], eribulin [37], ixabepilone [38–40]) and of new anti-HER2 targeted therapies (pertuzumab [41] and T-DM1 [42]) in advanced breast cancer treatment. Similar results from a parallel phase 2 study of olaparib monotherapy in recurrent ovarian, fallopian tube, or peritoneal cancers were reported by Audeh et al. In germline *BRCA1*/*2* mutation-positive patients, the objective response rate was 33 % [43]. It should be noted that in both trials, for the first time, a documented germline *BRCA* mutation was an enrollment criterion [34, 43].

Gelmon et al. [7] assessed safety and efficacy of olaparib as a single agent in HGSOC and TNBC in an important trial that also demonstrated the feasibility of pre- and post-treatment biopsies. While sustained responses were documented in HGSOC, no confirmed objective response was shown in TNBC, regardless of *BRCA*-mutation status, although 50 % of *BRCA*-mutation carriers had a greater than 30 % reduction in the target lesion. The authors speculated that the lack of evidence of efficacy in BRCA-associated breast cancers in this trial could be due to chance because of small sample size or population characteristics (heavily pretreated patients) [7].

Kaufman et al. [6] reported data of a phase 2 study (NCT01078662) of olaparib monotherapy in 298 patients with diverse recurrent cancers (mostly ovarian, breast, pancreatic, and prostate) and confirmed *BRCA1*/*2* mutations (a study design called “basket trial”). Breast cancer tumor response rate was 12.9 % in 62 patients, and 47 % of patients had disease stabilization for ≥8 weeks. The lower objective response rate in this study compared with previous studies [5, 34] could be due to the fact that the study population was more heavily pretreated than in other trials (mean of 4.6 prior chemotherapy regimens in the metastatic setting vs. 3 in Tutt et al. [6]).

When tested in ovarian cancer, PARPi showed efficacy regardless of *BRCA* status. In the previously cited Gelmon et al. [7] study, olaparib induced sustained responses in non-*BRCA* mutant HGSOC. Responses to olaparib were also observed in ovarian cancer patients with wild type or unknown *BRCA* status in a study of maintenance therapy after platinum-based chemotherapy [44] and in a study of olaparib plus cediranib [45]. Molecular studies suggested that up to 20 % of HGSOC lose *BRCA1* or *BRCA2* function through epigenetic events [46], thus expressing an HR-deficient phenotype with sensitivity to PARPi even in the absence of somatic/germline *BRCA* mutation.

Studies of veliparib monotherapy in metastatic breast cancer are currently in progress [47, 48]; data on veliparib efficacy as single agent in gynecological cancers are already available. Coleman et al. [49] reported data from a multicenter phase 2 study in BRCA-associated persistent or recurrent ovarian, fallopian tube, or primary peritoneal cancer: objective response rate to single agent veliparib was 26 % and progression-free survival at 6 months was 54 %, without significant difference between platinum-sensitive or platinum-resistant tumors.

#### PARPi in combination therapy

PARPi have been tested in the treatment of metastatic breast cancer in combination with multiple compounds in phase 1 and 2 studies [22]. Preclinical data showed that veliparib exerts remarkable synergic activity with other cytotoxic compounds [50]: in particular, veliparib enhanced temozolomide’s cytotoxic effect even in tumor types not typically responsive to temozolomide [51] with a good safety profile. Veliparib has been further clinically explored mainly as a part of combination therapy. In a phase 2 trial in BRCA-associated breast cancers, treatment with veliparib and temozolomide offered a response rate of 22 % and a clinical benefit rate of 50 % (defined as complete response, partial response, or stable disease) [52]. Efficacy was successively confirmed in a larger expansion cohort with patients previously treated with platinum compounds or PARPi [30].

Other combinations between PARPi and chemotherapy drugs have been proven effective in early clinical trials: the best results in terms of efficacy emerged from combination with cisplatin [23, 53] and carboplatin [54, 55], as well as topotecan [56], with response rates in BRCA-related breast cancers up to 73 % [23, 53]. Contrasting data about the safety of the combination therapy approach emerged from these studies. The combination topotecan-olaparib showed dose-limiting hematological adverse events at sub-therapeutic doses of olaparib [57]; in contrast, veliparib combinations have been better tolerated overall.

It is still not clear which is the best chemotherapeutic companion for a PARPi, and studies show that different PARPi may combine more or less efficiently with cytotoxic drugs with different mechanisms of action [58, 59]. The differences in synergistic effect between cytotoxic drugs and PARPi may be explained by PARPi mechanisms of action. Indeed, some PARPi exert their cytotoxic effect mainly suppressing PARPs’ catalytic activity (veliparib), while others more by trapping PARPs to DNA (olaparib, talazoparib, rucaparib, niraparib) [18]. It has been proposed that PARP trapping is synergistic with alkylating agents, while PARP catalytic inhibition synergizes with topoisomerase I inhibitors [58]. In preclinical models, proliferation of breast cancer cells is more potently suppressed when both mechanisms of PARP inhibition are present [18]. On the other hand, the higher toxicity of this class of PARPi may render them more toxic in combination with cytotoxic therapies.

### Ongoing studies in the metastatic setting

Ongoing randomized phase 3 studies of PARPi in metastatic breast cancer are limited to patients with documented *BRCA1*/*2* mutations (Table 4). Three parallel study designs will test oral PARPi monotherapy vs. physician’s choice single agent chemotherapy in breast cancer patients with PARPi-naive metastatic disease with germline *BRCA1*/*2* mutations: BRAVO (niraparib, NCT01905592 [60]), EMBRACA (talazoparib, NCT01945775 [61]), and OlympiAD (olaparib, NCT02000622 [62]). Finally, study NCT02163694 [63] will test the efficacy of veliparib versus placebo in combination with carboplatin and paclitaxel in HER2-negative metastatic or locally advanced, unresectable, BRCA-associated breast cancer.

**Table 4:** Ongoing and recruiting phase 2/3 studies

Study name (NCT)	Ref.	Phase	Setting	Investigational arm(s)	Comparator arm(s)	Primary endpoint	Study status
Olaparib							
OlympiAD	[62]	3	ADV	Olaparib monotherapy	Physician’s choice CT	PFS	R
(NCT02000622)							
OlympiA	[68]	3	ADJ	Olaparib monotherapy	Placebo	IDFS	R
(NCT02032823)							
Veliparib							
BROCADE	[96]	2	ADV	Veliparib +	Placebo +	PFS	R
(NCT01506609)				(Temozolomide) or	CBDCA + PTX		
				(CBDCA + PTX)			
(NCT02163694)	[63]	3	ADV	Veliparib +	Placebo +	PFS	R
				CBDCA + PTX	CBDCA + PTX		
Brightness	[74]	3	NADJ	(Veliparib + CBDCA) or (Placebo + CBDCA) + Neoadjuvant CT	Placebo + Neoadjuvant CT	pCR rate	R
(NCT02032277)							
Talazoparib							
EMBRACA	[61]	3	ADV	Talazoparib monotherapy	Physician’s choice CT	PFS	R
(NCT01945775)							
ABRAZO	[131]	2	ADV	Talazoparib monotherapy	Single arm study	ORR	R
(NCT02034916)							
(NCT02282345)	[75]	2	NADJ	Talazoparib monotherapy	Single arm study	Safety	R
Niraparib							
BRAVO	[60]	3	ADV	Niraparib monotherapy	Physician’s choice CT	PFS	R
(NCT01905592)							
Rucaparib							
(NCT00664781)	[132]	2	ADV	Rucaparib monotherapy	Single arm study	ORR, safety	NR
(NCT01074970)	[133]	2	ADJ	Rucaparib +	Cisplatin	2y-DFS	NR
				Cisplatin			

*ADJ* Adjuvant, *ADV* Advanced, *CBDCA* Carboplatin, *CT* Chemotherapy, *IDFS* Interval disease-free survival, *NADJ* Neoadjuvant, *NR* Not yet recruiting, *ORR* Objective response rate, *PFS* Progression-free survival, *PTX* Paclitaxel, *R* Recruiting, *2y-DFS* 2-year disease-free survival

Results from these studies are eagerly awaited and, if positive, will form the basis of applications for Food and Drugs Administration approval of PARPi for the treatment of metastatic BRCA-associated breast cancer. Approval will require an acceptable safety profile (see below) in a well-characterized and defined target population that currently lacks a specific targeted therapy. In 2014, both the European Medicines Agency and the Food and Drugs Administration [64, 65] granted accelerated approval to olaparib in high-grade serous ovarian, fallopian tube, and primary peritoneal cancer based on the results of two phase 2 trials [44, 66].

### Going beyond the metastatic setting

Conventionally, new antineoplastic drugs are tested as adjuvant treatments for breast cancer after solid data from phase 3 trials in the metastatic setting become available. In the case of PARPi, the remarkable activity of olaparib and veliparib in multiple phase 2 trials and their manageable toxicity profiles have led to trials of several PARPi in the adjuvant and neoadjuvant settings (Table 4). The adjuvant trial OlympiA is evaluating 1 year of the PARPi olaparib [67]. Data for the acceptability of olaparib given for extended periods of time come from a phase 2 study of single agent olaparib as maintenance therapy in platinum-sensitive ovarian cancer – median duration of treatment 206 days – but some patients stayed on the medication for years [44].

The OlympiA trial (NCT02032823 [68]) will assess the efficacy and safety of up to 12 months of olaparib versus placebo as adjuvant treatment in patients with germline *BRCA1*/*2* mutations and high-risk hormone receptor-negative HER2-negative primary breast cancer who have completed definitive local treatment and neoadjuvant or adjuvant chemotherapy. Eligibility criteria have recently been expanded to allow for enrollment of high-risk hormone receptor-positive patients. Randomization will be stratified by prior neoadjuvant versus adjuvant chemotherapy, and according to the use of prior platinum-based chemotherapy for breast cancer. The post-neoadjuvant treatment group will comprise patients in whom pathologic complete response was not achieved following at least six cycles of neo-adjuvant chemotherapy. The primary end point will be interval disease-free survival, and the secondary end points will be overall survival, distant disease-free survival, and the development of new primary invasive cancers.

Rucaparib is being tested in a phase 2 trial as adjuvant treatment for TNBC or *BRCA*-mutated HER2-negative breast cancers with residual disease after preoperative chemotherapy (NCT01074970) [69]; preliminary data presented at ASCO 2014 showed no improvement in 1-year disease-free survival with rucaparib plus cisplatin versus cisplatin alone in the intent-to-treat population; rucaparib did not add substantial toxicity to the cisplatin treatment [70].

The I-SPY2 study assesses sequential novel agents in the neoadjuvant treatment of breast cancer. In the I-SPY-2 trial assessing the addition of veliparib and carboplatin to standard neoadjuvant therapy in TNBC, an estimated 52 % pathologic complete response rate was observed in the experimental arm versus 26 % in the standard treatment arm [71]. In the cooperative group neoadjuvant trials GeparSixto and Alliance 40603, the addition of carboplatin to standard neoadjuvant chemotherapy increased pathological complete response rates in TNBC from 42.7 % to 53.2 % and from 41 % to 54 %, respectively [72, 73]. In GeparSixto, this effect is most evident in patients with germline *BRCA1*/*2* or *RAD51* mutations (the pathological complete response rate with carboplatin was 66.7 % versus 43.5 % without carboplatin). Participants are currently being accrued to a randomized three arms phase 3 trial that will test the efficacy of the addition of carboplatin plus veliparib, carboplatin alone, or placebo to standard neoadjuvant chemotherapy (Brightness Study – NCT02032277) [74].

A pilot phase 2 study of neoadjuvant talazoparib monotherapy in BRCA-associated breast cancer is ongoing at MD Anderson Cancer Center in Texas [75].

An interesting possibility for the future development of PARP inhibition in BRCA-related breast cancers has been raised by To et al. [76], who demonstrated a chemopreventive effect of veliparib and olaparib in delaying mammary tumor development in BRCA1-deficient mice. Data in this field are still too limited to speculate whether these findings could be translated to humans, but the concept of a chemopreventive drug active in a population at high risk of developing breast cancer is nonetheless intriguing. The future of PARPi in prevention is not clear at this time because of some chemotherapy-like toxic effects on bone marrow, in particular [22].

### Safety of PARPi

Toxicities of PARPi monotherapy appear to be similar to cytotoxic chemotherapeutic agents. Data from prominent phase 1 and 2 studies are summarized in Table 5: the most frequently reported adverse events in published studies are grade 1–2 nausea, vomiting, diarrhea, fatigue, headache, and anemia. The most common grade 3–4 toxicities were nausea, vomiting, and hematological toxicity, with anemia, lymphopenia, and thrombocytopenia being the most common dose-limiting toxicities in dose-finding studies [5, 34].

**Table 5:** Most common toxicities in studies of PARPi monotherapy in breast cancer patients

Compound	Adverse event (range of occurrence across studies)	
	Grade 1–2	Grade 3–4
Olaparib [5–7, 34, 134]	Nausea (32–58 %)	
	Fatigue (30–50 %)	
	Vomiting (11–34 %)	Nausea (4–15 %)
	Anorexia (12–27 %)	Fatigue (5–15 %)
	Anemia (5–25 %)	Anemia (11–15 %)
	Headache (22 %)	Vomiting (4–11 %)
	Diarrhea (11–18 %)	Thrombocytopenia (3 %)
	Taste alteration (13 %)	
Veliparib [135]	Dizziness (7 %)	–
	Nausea (7 %)	
	Dysgeusia (7 %)	
Talazoparib [32]	Fatigue (26 %)	
	Nausea (26 %)	
	Alopecia (grade 1 only, 26 %)	Neutropenia (8 %)
	Anemia (13 %)	Thrombocytopenia (8 %)
	Neutropenia (10 %)	Anemia (5 %)
	Flatulence (10 %)	
	Thrombocytopenia (3 %)	
Niraparib [31]	Anemia (48 %)	
	Nausea (42 %)	
	Thrombocytopenia (35 %)	Thrombocytopenia (15 %)
	Fatigue (34 %)	Anemia (10 %)
	Anorexia (25 %)	Fatigue (8 %)
	Neutropenia (24 %)	Neutropenia (4 %)
	Constipation (23 %)	
	Vomiting (19 %)	
	Insomnia (10 %)	
Rucaparib [33]^a^	Fatigue (30–39 %)	
	Nausea (27–30 %)	
	Diarrhea (13–20 %)	
	Vomiting (23 %)	
	Dizziness (17 %)	
	Anorexia (11 %)	

Grading according to Common Toxicology Criteria for Adverse Event

^a^Grade of reported adverse events not specified

Conversely, dose-limiting toxicities observed in trials of PARPi in combination with cytotoxic agents include primarily hematologic toxicities [77, 78]. These potentiated toxicities might restrict the future development of some olaparib-cytotoxic combinations [79]. However, using an intermittent schedule of PARPi administration instead of continuous dosing has proved effective in overcoming this limitation [23].

One major concern with drugs that inhibit DNA damage repair mechanisms is the risk of development of new primary malignancies. A small number of cases of myelodysplastic syndrome and acute myeloid leukemia have been described in PARPi studies, with an incidence of <1 % [22]. It is noteworthy that most patients had already been treated with DNA-damaging classic chemotherapeutic drugs, which *per se*, represents a risk factor for development of new malignancies. Nonetheless, the increased concentration of gammaH2AX (a marker of DNA damage [80–82]) in tissues of patients treated with PARPi implies an accumulation of DSB in normal tissues and thus could lead to an increased risk of cancer secondary to DNA damage [22], warranting a high level of attention when developing PARPi therapy, especially in the adjuvant setting.

### Resistance to PARP inhibition

As with most targeted therapies, cancers develop resistance to PARPi. All tumors that responded initially to treatment with PARPi have ultimately progressed. So far, three mechanisms of resistance to PARPi have been demonstrated, while two others have been hypothesized [83–85] (Table 6). The first of the three established mechanisms is the development of secondary mutations that restore BRCA functionality. Preclinical and clinical evidence indicates that genomic instability promoted by PARPi in HR-deficient cells may result in secondary mutations in the mutated *BRCA1* or *BRCA2* gene with restoration of functional protein expression and induction of PARPi resistance [86–88]. The second mechanism involves increased drug efflux with consequent reduction of intracellular PARPi concentrations. PARP1 knock-out cells show dramatic overexpression of P-glycoprotein [89]; PARP inhibition induces up-regulation of P-glycoprotein expression in an *in vivo* mammary tumor model [59]. The third mechanism of PARPi resistance is based on loss of p53 binding protein 1 (53BP1). *In vitro* and *in vivo* experiments showed that mutations causing loss of 53BP1 are able to restore the HR in *BRCA1*/*2* mutated cells, at least partially [90–92]: this “DNA damage repair rewiring” ultimately leads to reduced sensitivity to PARPi [93].

**Table 6:** Mechanisms of resistance to PARP inhibitors

Mechanism of resistance	Proposed explanation	HR restoration
Restoration of BRCA functionality	Secondary mutation in *BRCA1*/*2*	Potentially complete
DNA damage repair rewiring	Mutations in p53 binding protein 1	Partial
Increased drug efflux	Overexpression of P-glycoprotein	None
Increased activity of BRCA1/2 proteins	Increased stimulation of hypomorphic BRCA1/2 protein expression	Partial
Decreased PARP expression	Epigenetic silencing or increased turnover	None

*HR* Homologous recombination. For further details refer to text and [83–85]

Another hypothesized, but still unconfirmed, mechanism of resistance to PARPi at the time of this submission is the presence of *BRCA1*/*2* forms with low level of expression, but that can be enhanced in the presence of opportune stimuli (such as increase in DSB due to PARP inhibition) – so called hypomorphic *BRCA1*/*2* [84]. Furthermore, the hypomorphs may lead to the reduced formation of PARP-DNA complexes because of decreased PARP expression (for example, by epigenetic silencing of the gene or increased turnover of the protein) [85].

Some of the aforementioned mechanisms of resistance are shared between PARPi and platinum compounds [94], but the degree of overlap is not clear. For example, Audeh et al. [43] reported response to olaparib in ovarian cancer regardless of previous platinum sensitivity or resistance, while in the basket trial by Kaufman et al. [6], response rate to olaparib across breast cancer patients showed a trend in favor of patients without prior platinum exposure. However, platinum sensitivity can persist after resistance to PARPi develops [95]. It is notable that most of the ongoing studies of PARPi in advanced breast cancer exclude patients who had been previously treated with platinum compounds [61, 63, 96, 97] or who progressed on platinum-based chemotherapy regimens [60, 62].

The existence of resistance mechanisms can limit the clinical utility of PARPi; strategies to overcome acquired resistance are needed. For example, it has been shown that drugs able to block efflux pumps may revert their PARPi resistance [59]. Further, when PARPi resistance is due to restoration of BRCA-proficiency, induction of a BRCAness phenotype via CDK1 inhibition may render the tumor cells again susceptible to PARPi [98].

### Predicting response to PARPi

No established biomarker of response to PARPi is currently available. A candidate biomarker is the homologous recombination deficiency (HRD) score, which combines three different DNA-based metrics of genomic instability that are highly associated with *BRCA1*/*2* mutational status or predictive of sensitivity to platinum chemotherapy [99]; Richardson et al. [100] demonstrated that the HRD score is able to identify patients with breast tumors with underlying HR deficiency (including *BRCA1*/*2* non-mutated tumors) that benefit from neoadjuvant platinum therapy. In PrECOG 0105, high HRD scores identified patients with a higher likelihood of achieving pathological complete response to platinum-based neoadjuvant chemotherapy [101]. However, data from the GeparSixto study showed a statistically significant increase in pathological complete response rates in patients with high HRD score; the benefit was observed irrespective of *BRCA1*/*2* status (mutated versus intact) [102]. These results could not be replicated in the advanced setting, although the fact that the HRD assay was performed on primary tumor specimens rather than metastatic samples may have limited its ability to predict responsiveness to carboplatin in metastatic breast cancers (TNT trial) [103]. The value of HRD score in predicting response to therapy is being prospectively tested in both neoadjuvant and advanced settings using platinum compounds [104] and PARPi [97, 105], respectively. Other promising biomarkers are the assessment of PARP activity through measurement of poly(ADP-ribose) levels [93, 106], the evaluation of HR proficiency through the formation of nuclear RAD51 foci [107, 108], the presence of miRNAs involved in the regulation of BRCA proteins (such as miR-182) [109], and the evaluation of levels of 53BP1 expression [17, 93].

### Strategies to expand PARPi application to BRCA-proficient breast cancers

Theoretically, PARPi activity could be expanded to breast cancers without *BRCA1*/*2* mutations; several preclinical experiments support this possibility by focusing on the impairment of the HR pathway. PTEN [110] and ATM [111, 112] deficiencies correlate with sensitivity to PARPi both *in vitro* and *in vivo*; moreover, CDK1 inhibition [98] and histone deacetylase inhibition [113] have been shown to efficiently sensitize BRCA-proficient cells to PARPi *in vitro* and, in animal models, *in vivo*. A phase 1 study is ongoing in patients with solid tumors testing the association of veliparib, a selective CDK inhibitor (dinaciclib) and carboplatin: an expanded cohort of BRCA-proficient tumors is planned [114]. Unfortunately, no validated biomarker of HR dysfunction other than germline *BRCA1*/*2* mutations is currently available.

Alterations in the HR pathway different from *BRCA1*/*2* mutations may determine an HR-deficient phenotype similar to BRCA-deficient tumor (namely, BRCAness) [20]. Such alterations include *BRCA1*/*2* suppression (for example, by promoter methylation) or mutations in genes encoding other proteins involved in HR (such as *PTEN*, *FANCF*, *RAD51*, *ATM*, and *CDK1*) [20, 28, 110]. In line with this hypothesis, talazoparib will be tested in *BRCA1*/*2* wild type breast cancer with high HRD score or deleterious germline or somatic mutation implicated in the HR pathway [97].

Other options to exploit PARP inhibition in BRCA-proficient breast cancers currently under investigation (mainly in cell lines and animal models, but also in clinical trials) include PI3K inhibition [115, 116] and TGFβ activation [117]. Preliminary positive data of clinical efficacy of PARPi/PI3K inhibitors in *BRCA* wild type ovarian and breast cancer have been presented by Matulonis et al. [118] at the 2015 American Association for Cancer Research Annual Meeting.

## Conclusions

PARP inhibition is a promising strategy for the treatment of breast cancer associated with germline *BRCA1*/*2* mutations and papillary serous ovarian cancers. Efficacy data from phase 1 and 2 studies showed encouraging objective response rates with acceptable toxicity profiles for PARPi monotherapy. The initial data are consistent with those of other targeted therapies in identifiable subsets of tumors. There is great excitement about the ongoing phase 3 trials in the metastatic, adjuvant, and neoadjuvant settings.

However, other questions apart from clinical efficacy need to be addressed before PARPi will become part of clinical practice. For example, the long-term effects of continuous administration of this class of drugs are not yet fully characterized: will prolonged exposure to PARPi confer increased risk of hematological toxicity or development of new primary malignancies? This is a concern of particular importance in the adjuvant setting. Increasing use of platinum in early triple-negative disease may influence the way PARPi are used given the overlapping mechanisms of action and resistance.

New strategies are being examined to expand the application of PARPi in BRCA-associated cancers beyond breast and ovarian, and in some sporadic tumors. PARPi should be more fully studied in ER-positive BRCA-associated tumors as well. PARPi appear likely to assume an important role in the management of patients with BRCA-associated tumors, and possibly in other carefully defined tumor subsets as well.

## Abbreviations

53BP1: p53 binding protein 1
BER: Base excision repair pathway
HGSOC: High-grade serous ovarian cancer
HR: Homologous recombination
HRD: Homologous recombination deficiency
PARPi: PARP inhibitors
PARPs: Poly(ADP-ribose) polymerases
SSBR: Single strand break repair
TNBC: Triple-negative breast cancers
